# Machine Learning Can Predict the Probability of Biologic Therapy in Patients with Inflammatory Bowel Disease

**DOI:** 10.3390/jcm11154586

**Published:** 2022-08-05

**Authors:** David Schöler, Karel Kostev, Maximilian Peters, Cosmin Zamfir, Agnieszka Wolk, Christoph Roderburg, Sven H. Loosen

**Affiliations:** 1 Clinic for Gastroenterology, Hepatology and Infectious Diseases, University Hospital Düsseldorf, Medical Faculty of Heinrich Heine University Düsseldorf, Moorenstrasse 5, 40225 Düsseldorf, Germany; 2 Epidemiology, IQVIA, 60549 Frankfurt, Germany; 3 Real World Insights, IQVIA, 60549 Frankfurt, Germany; 4 Data Science & Advanced Analytics, IQVIA, 60549 Frankfurt, Germany

**Keywords:** inflammatory bowel disease, biologics, machine learning, Light Gradient Boosting Machine

## Abstract

Background: Inflammatory bowel disease (IBD) is of high medical and socioeconomic relevance. Moderate and severe disease courses often require treatment with biologics. The aim of this study was to evaluate machine learning (ML)-based methods for the prediction of biologic therapy in IBD patients using a large prescription database. Methods: The present retrospective cohort study utilized a longitudinal prescription database (LRx). Patients with at least one prescription for an intestinal anti-inflammatory agent from a gastroenterologist between January 2015 and July 2021 were included. Patients who had received an initial biologic therapy prescription (infliximab, adalimumab, golimumab, vedolizumab, or ustekinumab) were categorized as the “biologic group”. The potential predictors included in the machine learning-based models were age, sex, and the 100 most frequently prescribed drugs within 12 months prior to the index date. Six machine learning-based methods were used for the prediction of biologic therapy. Results: A total of 122,089 patients were included in this study. Of these, 15,824 (13.0%) received at least one prescription for a biologic drug. The Light Gradient Boosting Machine had the best performance (accuracy = 74%) and was able to correctly identify 78.5% of the biologics patients and 72.6% of the non-biologics patients in the testing dataset. The most important variable was prednisolone, followed by lower age, mesalazine, budesonide, and ferric iron. Conclusions: In summary, this study reveals the advantages of ML-based models in predicting biologic therapy in IBD patients based on pre-treatment and demographic variables. There is a need for further studies in this regard that take into account individual patient characteristics, i.e., genetics and gut microbiota, to adequately address the challenges of finding optimal treatment strategies for patients with IBD.

## 1. Introduction

Inflammatory bowel disease (IBD) is of high medical and socioeconomic relevance. There are around 6–8 million cases of IBD worldwide, with prevalence rising in industrialized countries [1]. Moderate and severe disease courses often require the use of immunosuppressive drugs, including azathioprine and TNF-alpha-, JAK1/3-, and IL12/23-inhibitors, to maintain remission. A variety of risk factors for the development of IBD have been identified in the past [2], such as genetic factors, e.g., mutations in *NOD2* for Crohn’s disease, or changes in the gut microbiota [3]. However, the pathogenesis of IBD is still largely unknown, and there is a lack of predictive criteria for a clinical response to a particular therapy. In clinical routine, many years of therapy with alternating therapy regimens are often needed to find the appropriate substances for individual patients. Depending on the severity of the disease, this is also associated with an increased risk of cancer, especially in individuals with ulcerative colitis, and the need for surgery, which represents a significant morbidity/mortality risk and also significantly reduces patient quality of life.

Recently, machine learning (ML) algorithms have been increasingly used to support clinical decision making, e.g., in predicting the mortality of septic patients via extreme gradient boosting (XGBoost; [4]), or in the assessment of myocardial ischemia using angiography with a Light Gradient Boosting Machine (GBM) [5]. The aim of this study was to evaluate ML-based methods for the prediction of biologic therapy in patients with Crohn’s disease and ulcerative colitis using a large prescription database.

## 2. Materials and Methods

### 2.1. Data Sources

The present retrospective cohort study was based on the IQVIA LRx database [6]. This database comprises about 80% of prescriptions reimbursed by statutory health insurance funds in Germany. Data are available at the patient level and include information on patient age and sex. In accordance with data privacy legislation, all patient information is fully anonymized by the data provider. Each prescription is available with the full product information (e.g., brand, substance, package size, and product form) and dates dispensed. The database does not contain diagnoses or details of laboratory tests. The LRx database has been used in various previous studies [7]. As the LRx database contains prescription information, but no diagnosis information, data from the Disease Analyzer database (IQVIA) were used for the present study to define Crohn’s disease (CD) and ulcerative colitis (UC) cases based on the prescribed therapy. The Disease Analyzer database covers around 3% of primary-care practices in Germany. This database includes demographic, diagnosis, and prescription data obtained in an anonymized format from computer systems used in general and specialized practices. Previous research has shown that this is representative of primary-care practices in Germany [8].

### 2.2. Study Population and Outcome

Using data from the Disease Analyzer database, we first compared prescribed therapies in patients with CD/UC attending gastroenterologist practices with those in patients without CD/UC. The most significant difference was observed for intestinal anti-inflammatory agents (ATC: A07E excluding vedolizumab), with prescription rates of 80% in CD/UC patients and 2.0% in non-CD/UC patients. Finally, we included all patients from the LRx database with at least one prescription for an intestinal anti-inflammatory agent from a gastroenterologist between January 2015 and July 2021. Patients who received an initial biologic therapy prescription (infliximab, adalimumab, golimumab, vedolizumab, or ustekinumab) were categorized as the “biologic group”. These patients were classified as early adopters (patients whose first biologics prescription was issued at least 180 days prior to the last record in the database) and late adopters (patients whose first biologics prescription was within the time period of 0–180 days before the last record in the database). The date of the first prescription for biologics was considered the index date. Patients who did not receive any biologics during the study period were classified as the “non-biologic group”. For these patients, the last prescription for any drug documented in the database was considered the index date. One further criterion for inclusion was an observation time of at least 18 months (prior to the last database record).

### 2.3. Potential Predictors and Statistical Analyses

The objective of the ML model was to differentiate between patients who received biologics and those who did not, and to identify key predictors of biologic therapy initiation. Potential predictors included in the machine learning-based models corresponded to prescription data obtained prior to the index date. More than 400 different drugs prescribed within 12 months prior to the index date were found. The model took into account the 100 most frequently prescribed drugs as well as patient age and sex, and prescriptions were normalized to the total number of prescriptions per patient.

Six machine learning-based methods for the prediction of biologic therapy were tested: Light Gradient Boosting Machine (LGBM) [9], linear support vector classification (SVC) [10], logistic regression [11], cat boost [12], competitive gradient descent (CGD) [13], and random forest [14].

Training of the ML systems was completed using data from non-biologics patients and early adopters. The dataset was split into two sets: 80% for training and 20% for testing. The LRx database used provides a snapshot of each patient’s prescription history. It was therefore possible that a patient had not yet received biologics but would receive them in the future. We tested whether a subset of the misclassified non-biologics patients, i.e., false-positive patients, might be future biologics patients. This experiment was performed by excluding late adopters from the training set and testing them separately. To compensate for the imbalanced dataset, the class weight for the biologics patients was set to the ratio between non-biologics and biologics patients.

## 3. Results

### 3.1. Characteristics of the Study Sample and Incidence of Biologic Therapy

A total of 122,089 patients were included in this study. The mean (standard deviation) age of the study sample was 51.3 (SD: 18.5) years, and 55.7% of patients were female. The five most frequently prescribed therapies within 12 months prior to the index date were mesalazine (55.9%), budesonide (35.2%), pantoprazole (27.5%), metamizole (27.0%), and prednisolone (24.5%). As intestinal anti-inflammatory agents were used for the definition of the study population, they were not considered a co-therapy. Of the 122,089 IBD patients, 15,824 (13.0%) received at least one prescription for a biologic drug. Biologics patients were younger (43.9 (SD: 15.8) years) than non-biologics patients (52.9 (SD: 17.5) years). The proportion of women was slightly lower among the biologics patients (53.6% vs. 56.5%).

### 3.2. Performance of the Biologic Therapy Prediction Models

**Figure 1:**
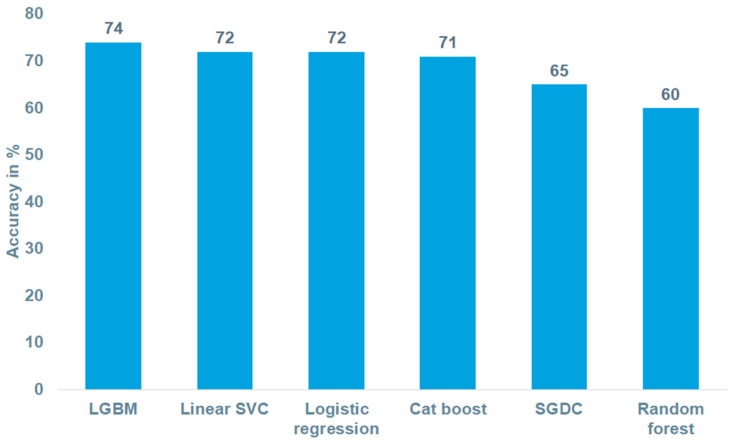
Accuracy of algorithms evaluated.

Figure 1 outlines the performance of the biologic therapy prediction models. The performance of LGBM (accuracy = 74%) was highest, followed by LSVC (72%) and logistic regression (72%).

Abbreviations: LGBM—Light Gradient Boosting Machine; SVC—Support Vector Classifier; SGDC—Stochastic Gradient Descent Classifier. The F1 score was calculated using the training dataset.

**Figure 2:**
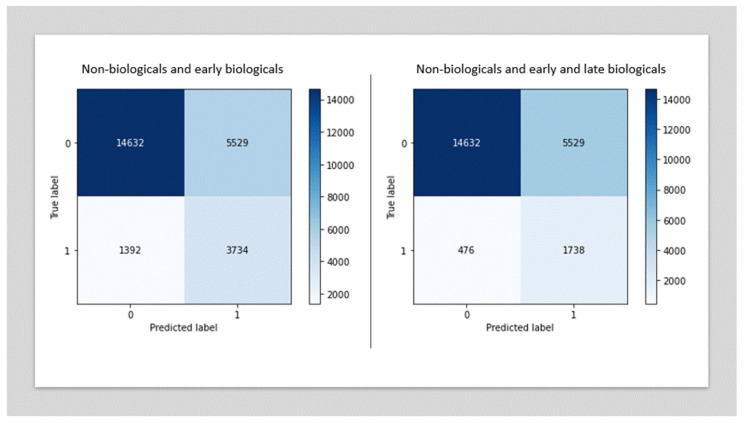
Predicted and true biologics users (LGBM classifier).

In terms of sensitivity, the LGBM model was able to correctly identify 78.5% of the biologics patients and 72.6% of the non-biologics patients in the testing dataset (Figure 2). The majority of the patients who were classified as biologic in the training dataset but who had not received biologics at this point were correctly classified as biologics patients in the test dataset containing late adopters. The chosen algorithm was therefore able to identify patients for whom traditional therapies were failing and for whom biologics would be used in the future.

The prediction of biologics patients was slightly less accurate when late adopters were included (72.9%) (Figure 2).

### 3.3. Most Important Variables Predicting the Probability of Biologic Therapy

Table 1 displays the therapies with the largest differences in proportions between patients with and without biologic therapy. Calcium products (12.3% vs. 3.9%), immunosuppressants (mainly azathioprine, 33.3% vs. 11.0%), iron products (24.7% vs. 8.6%), and oral corticosteroids (71.1% vs. 26.1%), as well as antidiarrheal microorganisms (4.7% vs. 2.0%), were the therapy classes with the biggest differences.

**Figure 3:**
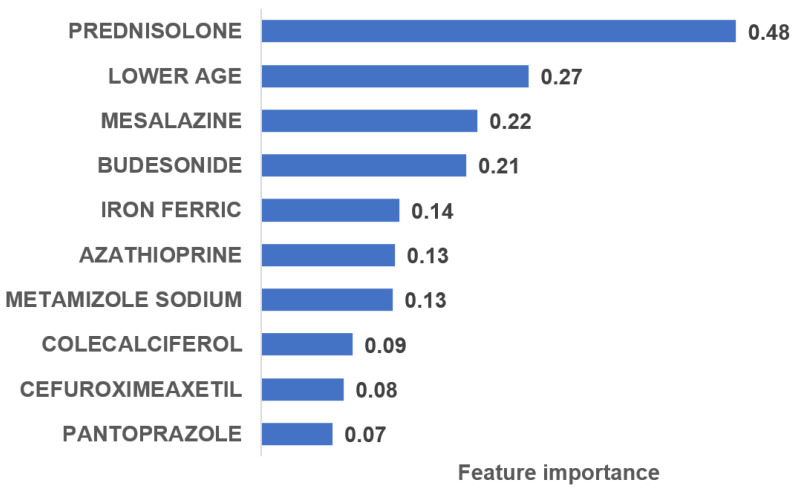
Feature importance in the LGBM model (top 10 variables).

Figure 3 shows the feature importance of the variables from the LBGM model. The most significant variable was prednisolone, followed by lower age, mesalazine, budesonide, and ferric iron. These variables were followed by the use of azathioprine, metamizole sodium, colecalciferol, cefuroxime axetil, and pantoprazole. Of note, the use of prednisolone, budesonide, and azathioprine is part of first-line therapy in the treatment of IBD. A much higher value was found for prednisolone when compared to the other variables in the top 10.

## 4. Discussion

In the present study, six ML-based models were tested to determine their accuracy in the prediction of biologic therapies in a large cohort of more than 120,000 IBD patients. All the ML models tested had a high level of accuracy, with LGBM achieving the highest F1 values. The prescriptions of corticosteroids, mesalazine, and ferric iron, as well as lower age, were the variables with the highest feature importance.

Our data suggest that biologics tend to be considered more often for the treatment of younger patients. Some studies have demonstrated that clinical features and the extent of disease in IBD patients appear to be more severe with younger age [15,16]. As biologics are usually prescribed for patients with severe disease, this may explain the role of younger age as one of the most significant predictors. In their large study completed in South Korea, Choi et al. reported that the odds of receiving biologic drugs were 2.3 times higher in the early-onset group than in the late-onset group [17].

A high proportion of patients received oral corticosteroids, intestinal corticosteroids, and other immunosuppressants (mainly azathioprine), but also iron prior to biologic therapy, and these therapies were important predictor features in the ML model. The prescription of these drugs is common practice. For example, vedolizumab and ustekinumab have been approved for the treatment of patients with moderate to severe UC or CD who exhibit an inadequate response, loss of response, or intolerance to corticosteroids and immunosuppressives [18].

Iron is usually given to patients with anemia, which is a common extraintestinal manifestation of IBD. Patients with IBD commonly have iron-deficiency anemia due to chronic blood loss and impaired iron absorption as a result of tissue inflammation [19]. This complication also demonstrates the severity of IBD, which can impact the patient’s chances of receiving a biologic drug.

Vitamin D (colecalciferol) was one of the top 10 most commonly prescribed medications in the ML model. Vitamin D deficiency is common in patients with active IBD [20] and is linked to disease activity, more frequent relapses, and higher postoperative recurrence [21]. A further symptom of severe IBD is pain, which is a long-standing problem for the majority of the patients affected [22]. Metamizole, which was also one of the top ten medications in the ML model, is an analgesic often prescribed in IBD patients.

Although cefuroxime axetil also ranked among the top ten most common medications in the model, it was found to have lower future significance in predicting the prescription of biologics. This antibiotic drug is sometimes used to treat bacterial infections that may arise due to complications of IBD and which may cause fistulas [23].

This study is subject to a number of limitations. First, patients were selected based on the prescription of intestinal anti-inflammatory drugs, which presents the possibility of selection bias. Although the administration of these drugs reflects the common guidelines for the treatment of patients with CD/UC, a number of patients do not receive them, e.g., because of intolerance. Second, the diagnoses of CD and UC were based on database queries [6,8] and were not standardized by the same clinical criteria, i.e., by the use of a positive histology. Moreover, in the LRx database, no information about diagnoses was available and no stratification into CD and UC was possible. Third, the database did not include data on therapy duration and daily dose for the majority of patients. Fourth, the precision of the ML models was not tested in different subgroups while taking into account the severity of the disease. Fifth, the external test sets were not available. Sixth, the retrospective analysis only shows associations and does not show any causal relationships.

However, as other working groups suggest [24,25], our study shows that in combination with big data, ML approaches can help improve the treatment of IBD patients in the future.

In summary, this investigation studied the feasibility of ML-based models in predicting future biologic therapy in IBD patients based on pre-treatment and demographic variables. Further studies are required that take into account individual patient characteristics, i.e., genetics and gut microbiota [2,3], to adequately address the challenges of finding optimal treatment strategies for patients with IBD.

## Figures and Tables

**Table 1:** Therapies with largest proportional differences between patients with and without biologic therapy.

Therapy Class	Proportion among Biologics Patients in % (n = 15,284)	Proportion among Non-Biologics Patients in % (n = 106,265)	Prevalence Ratio (Biologics/Non-Biologics)	*p*-Value
Calcium	12.3	3.9	3.2	<0.001
Other immunosuppressants *	33.3	11.0	3.0	<0.001
Iron	24.7	8.6	2.9	<0.001
Oral corticosteroids	71.1	26.1	2.7	<0.001
Antidiarrheal microorganisms	4.7	2.0	2.4	<0.001
Intestinal corticosteroids	64.2	33.4	1.9	<0.001
Vitamin D	20.1	10.9	1.8	<0.001
Oral fluoroquinolones	10.4	6.9	1.5	<0.001
Gastroprokinetics	6.9	4.7	1.5	<0.001
Antitussives	6.8	4.7	1.4	<0.001
PPI	54.0	41.4	1.3	<0.001

* Mainly azathioprine.

## Data Availability

The data that support the findings of this study are available on request from the corresponding author.
